# A Cost-Effective and Easy-to-Fabricate Conductive Velcro Dry Electrode for Durable and High-Performance Biopotential Acquisition

**DOI:** 10.3390/bios14090432

**Published:** 2024-09-06

**Authors:** Jun Guo, Xuanqi Wang, Ruiyu Bai, Zimo Zhang, Huazhen Chen, Kai Xue, Chuang Ma, Dawei Zang, Erwei Yin, Kunpeng Gao, Bowen Ji

**Affiliations:** 1 Unmanned System Research Institute, Northwestern Polytechnical University, Xi’an 710072, China; gj18831723531@mail.nwpu.edu.cn (J.G.); xuanqi@mail.nwpu.edu.cn (X.W.); Rybai@mail.nwpu.edu.cn (R.B.); zhangzimo@mail.nwpu.edu.cn (Z.Z.); huazhenchen@mail.nwpu.edu.cn (H.C.); xuekai@mail.nwpu.edu.cn (K.X.); 2 National Key Laboratory of Unmanned Aerial Vehicle Technology, Integrated Research and Development Platform of Unmanned Aerial Vehicle Technology, Northwestern Polytechnical University, Xi’an 710072, China; 3 Ministry of Education Key Laboratory of Micro and Nano Systems for Aerospace, School of Mechanical Engineering, Northwestern Polytechnical University, Xi’an 710072, China; 4 Defense Innovation Institute, Academy of Military Sciences (AMS), Beijing 100071, China; ustb_machuang@163.com; 5 Intelligent Game and Decision Laboratory, Beijing 100071, China; 6 Tianjin Artificial Intelligence Innovation Center (TAIIC), Tianjin 300450, China; 7 Department of Rehabilitation Medicine, Beijing Tiantan Hospital, Capital Medical University, Beijing 100070, China; zangdawei@bjtth.org; 8 College of Information Science and Technology, Donghua University, Shanghai 201620, China

**Keywords:** conductive Velcro electrode, hook structure, biopotential detection, gesture recognition

## Abstract

Compared with the traditional gel electrode, the dry electrode is being taken more seriously in bioelectrical recording because of its easy preparation, long-lasting ability, and reusability. However, the commonly used dry AgCl electrodes and silver cloth electrodes are generally hard to record through hair due to their flat contact surface. Claw electrodes can contact skin through hair on the head and body, but the internal claw structure is relatively hard and causes discomfort after being worn for a few hours. Here, we report a conductive Velcro electrode (CVE) with an elastic hook hair structure, which can collect biopotential through body hair. The elastic hooks greatly reduce discomfort after long-time wearing and can even be worn all day. The CVE electrode is fabricated by one-step immersion in conductive silver paste based on the cost-effective commercial Velcro, forming a uniform and durable conductive coating on a cluster of hook microstructures. The electrode shows excellent properties, including low impedance (15.88 kΩ @ 10 Hz), high signal-to-noise ratio (16.0 dB), strong water resistance, and mechanical resistance. After washing in laundry detergent, the impedance of CVE is still 16% lower than the commercial AgCl electrodes. To verify the mechanical strength and recovery capability, we conducted cyclic compression experiments. The results show that the displacement change of the electrode hook hair after 50 compression cycles was still less than 1%. This electrode provides a universal acquisition scheme, including effective acquisition of different parts of the body with or without hair. Finally, the gesture recognition from electromyography (EMG) by the CVE electrode was applied with accuracy above 90%. The CVE proposed in this study has great potential and promise in various human–machine interface (HMI) applications that employ surface biopotential signals on the body or head with hair.

## 1. Introduction

In the past few decades, epidermal electronics have exhibited enormous potential in personal medical monitoring, energy harvesting, and wearable displays [1,2,3]. There has been extensive research on durable, stable, and wearable devices for the long-term monitoring and transmission of electrophysiological (EP) signals, like electro-myogram (EMG) [4], electrocardiogram (ECG) [5], electroencephalogram (EEG) [6], and electro-oculogram (EOG). Clinically, these diverse EP signals can offer useful information. Among them, surface electromyography (sEMG) signals can function as driving signals for actuating robots or prostheses and are also of great importance for HMI [7,8]. In studies, researchers can reduce the impedance between electrodes by cleaning the skin and filling electrolyte gel between the scalp and the electrodes, but these methods and technologies may cause discomfort to the subjects and require high time costs [9]. The dry electrode makes the electrode fit to the scalp under the action of mechanical force [10]. Electrolytes such as gel are not required between the scalp and the electrode point. The dry electrode directly contacts the scalp without gel filling, which greatly reduces the time and cost of data recording preparation [11]. While enhancing flexibility [12], the acquisition system can also mitigate the potential for recording more noise by amplifying the signal [13]. Dry electrodes provide the possibility of long-term continuous recording and can be applied to aging [14], infant, and patient populations, as well as sports, driving, classroom, and marketing environments [15].

Currently, Ag/AgCl electrodes are the most frequently employed type of dry electrodes for recording sEMG signals [16,17]. They have a large size and strong adhesion. This causes users to experience pain when peeling them off. Additionally, they are single-use and can lead to skin irritation and redness after usage [18,19]. Consequently, in clinical settings, significant efforts have been made to find alternative new materials and electrodes for long-term EP signal monitoring to address the drawbacks of Ag/AgCl electrodes and meet wearable requirements [20,21,22,23,24]. An on-skin electrode for monitoring EP signals [25], namely, the microneedle dry electrode, can be used to measure high-quality signals [26,27]. However, it is invasive to human skin and may pose a risk to human health [28]. Moreover, the fabrication process of microneedle dry electrodes requires high-precision photolithography and clean room facilities, which is costly [29,30,31]. Recently, dry metal discs and combs were introduced to collect electromyographic signals [32,33,34,35]. They have advantages such as electrode-to-skin contact impedance stability, signal intensity stability, and a certain electrode size. Nevertheless, their weight and flexibility may not be suitable for long-term monitoring and wearable applications [36]. This led to the development of fabric electrodes, such as the copper-plated textile fabric EMG electrode and knitted soft textile electrodes made of nylon, conductive fibers, spandex, and polypropylene for EMG monitoring [37]. As smart textiles for health monitoring are emerging rapidly, it is beneficial to develop textile-based biopotential sensors for active control of human health while maintaining the comfort and bulk properties of the textile to make it more user-friendly [38]. Velcro, as a type of textile, appears in every aspect of our lives. The hook of the Velcro has a large air raid around it [39,40,41,42], which can penetrate through hair to collect biopotentials [43]. The shape of the Velcro is very similar to that of a claw electrode, but due to the bendable hook hair of the Velcro, it has better flexibility and stronger wearing comfort compared to the claw electrodes. At the same time, the price of Velcro is relatively cheaper than other electrodes, and the future market is promising [44].

In this paper, we report a strategy to fabricate a novel CVE with low impedance, high signal-to-noise ratio, strong water resistance, and compression resistance for bioelectrical signal acquisition. The CVE consists of Velcro, conductive silver paste, and an electrode buckle. The electrode is produced in one step, forming a uniform and reliable conductive coating on the surface of the electrode. Electrochemical and signal quality tests have been performed to verify the conductivity of the CVE. The water resistance and compression resistance were verified by a washing test and a compressing test. In contrast to traditional dry electrodes, impedance tests and surface electromyography measurements were carried out on the subject’s muscle. The outcomes indicate that the CVE is reliable and durable when worn.

## 2. Materials and Methods

### 2.1. Materials

Velcro was purchased from Hengtong Weiye Special Fabric Technology Co., Ltd., Qingdao, China, and conductive silver paste was purchased from Jingzhe Technology Co., Ltd., Shenzhen, China. By soaking Velcro in conductive silver paste, the Velcro gains conductivity. The oven used for curing the conductive silver paste was purchased from Hengnuo Lixing Technology Co., Ltd., Beijing, China. The all-solid-state nanosecond laser used for cutting the Velcro was purchased from Huari Precision Laser Co., Ltd., Wuhan, China, with the model number Poplar2-355-5AZ1; the nanosecond laser cutting machine was purchased from Langrui Laser Technology Co., Ltd., Xi’an, China. When cutting the Velcro, the laser parameters were set to a frequency of 200 kHz, pulse width of 200 ms, current of 10 A, and marking speed of 100 mm/s, and each Velcro was processed 25 times.

### 2.2. Impedance Measurement

The electrochemical impedance spectroscopy (EIS) for skin contact impedance was measured through an electrochemical workstation (CHI600E, Chenhua, Shanghai, China). For measuring the skin contact impedance of the CVE, a three-electrode system was employed [45]. The working electrode was chosen as CVE and AgCl electrodes for comparison purposes. The counter and reference electrodes were commercial AgCl electrodes. The working electrode and the counter electrode were positioned on the arm with a center interval of 5 mm, and the reference electrode was placed on the elbow [46]. The AC voltage amplitude was 5 mV, sweeping from 1 Hz to 100 kHz [47]. Each experiment was performed at least five times. The AgCl electrode was tested using an electrochemical workstation, which was purchased from Guanlong Medical Technology Co., Ltd., Dongguan, China. The claw electrode and silver cloth electrode were purchased from Yiyiji Technology Co., Ltd., Suzhou, China.

### 2.3. SNR Calculation

The signal-to-noise ratio (SNR) of the sEMG signals for all the electrodes was computed using the following equation.

(1) $${\mathrm{SNR}}_{db}={10\log_{10}(\frac{A_{\mathrm{signal}}}{A_{\mathrm{noise}}})}^{2}=20\log_{10}\frac{A_{\mathrm{signal}}}{A_{\mathrm{noise}}}$$

Here, *A_signal_* denotes the root-mean-square (RMS) of the sEMG signals. Meanwhile, *A_noise_* represents the RMS of noise recorded in calm states and is also regarded as the baseline of noise. The biopotential acquisition equipment was purchased from OpenBCI (New York, NY, USA). The sampling rate of the electromyographic acquisition equipment was set to 250 Hz [48], the delay time was set to 20 ms, and the sampling protocol was set to TCP protocol. The data transmission adopted Bluetooth transmission [49]. During eye electrical collection, the working electrode was placed 1–2 cm above the eyebrows, the counter electrode was placed 1–2 cm below the eyes, and the reference electrode was placed on the earlobe. During electrocardiogram collection, the working electrode was attached to the heart, while the counter electrode and reference electrode were attached to both sides of the stomach [50]. Before collecting electrical signals, we cleared the desktop or moved the collection equipment to an open area.

### 2.4. Water Resistance Test and Compression Resistance Testing

To investigate the washability performance of the electrodes, the samples were washed in a beaker. The printed electrodes were washed with 4 g/L nonionic detergent at 40 °C for 30 min according to the standard method (ISO 105-C06-A1S, 2010) [51]. The speed of the magnet was 400 rpm. The sample was naturally dried at room temperature [52].

We then verified the anti-compression performance of the CVE. The mechanical tensile platform used in the compression test of the Velcro was purchased from Care Measurement & Control Technology Co., Ltd., Tianjin, Chia. The load range during the experiment was set to 0.5–10 N for 50 cycles.

### 2.5. Gesture Recognition Method

Four gestures covering various degrees of freedom of wrist activity were selected as the target gestures: palm open, palm closed, wrist adduction, and wrist extension [53]. The working electrode and counter electrode were attached to the forearm, and the reference electrode was attached to the elbow. Each gesture was collected 60 times at a speed of 2 s. The constructed convolutional neural network CNN consisted of two convolutional blocks, each including the CONV layer, BN layer, RELU layer, and MAX pooling layer. The first convolutional kernel size was 2×1, generating 64 convolutions, and the second convolutional kernel size was 2×1, generating 128 convolutions. The learning rate during training was set to 0.001. The system running the robot simulation program was Ubuntu 20.04 [54].

## 3. Results

### 3.1. Fabrication Processes of the CVE

**Figure 1:**
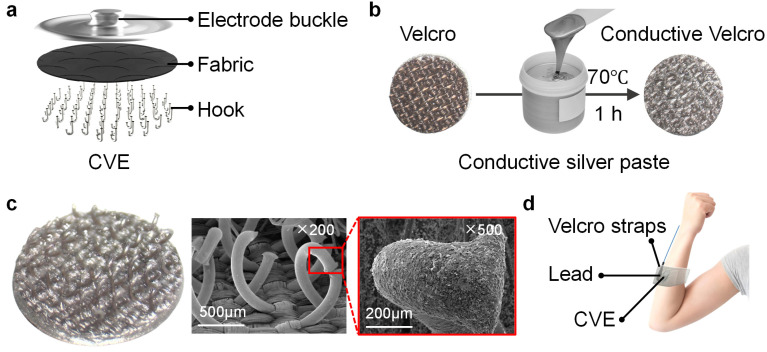
Fabrication and wearing method of the conductive Velcro as an electrode. (**a**) The internal structure of CVE; (**b**) the manufacturing process of CVE; (**c**) the microstructure of CVE; (**d**) the wearing method of CVE.

The internal structure of the CVE is shown in Figure 1a. The electrode can be divided into three parts from top to bottom: the electrode buckle, fabric, and hook. The fabric and hook are a whole unit. The preparation process of the CVE is shown in Figure 1b. Firstly, the Velcro was cut into small circular pieces with a diameter of 1 cm using the laser cutting machine. Then, the Velcro was soaked in a conductive silver paste solution to completely cover the surface of the Velcro. Finally, the electrode buckle and Velcro were pasted together using the viscosity of the conductive silver paste. They were heated in an oven for 1 h (70 °C) until the conductive silver paste was completely cured. The microstructure of the electrode hook hair of the conductive Velcro is shown in Figure 1c. The left figure is the electrode. The physical picture shows that the surface of the Velcro is completely wrapped in conductive silver paste [55], and the middle image is a magnified photo (200 times) of the CVE hook hair taken with a scanning electrode. When magnified 500 times, it can be seen that the hook hair surface of the conductive Velcro is covered with silver particles, which greatly enhances the conductivity of the Velcro electrode. The wearing method of the CVE is shown in Figure 1d. The hook hair of the CVE was attached to the skin surface, and the electrode buckle on the back of the CVE was connected to the external lead. The external lead and CVE were fixed to the skin surface by a bandage. When the electrode is working, the biopotential on the skin surface is transmitted to the external device through the hook, fabric, electrode buckle, and lead, which is then further analyzed.

### 3.2. Microstructure of the CVE

**Figure 2:**
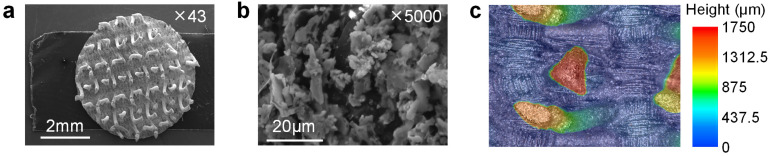
Microstructure of the CVE. (**a**) Scanning electron microscopy (SEM) of the electrode surface at 43 times magnification; (**b**) SEM of the conductive silver on the hook hair surface at 5000 times; (**c**) ultra-depth microscope picture of the hook microstructure on the CVE surface.

The CVE has a unique structure similar to claw electrodes, but their hooked hair structure is softer and more elastic than the claws on claw electrodes. Thus, the CVE can effectively reduce the discomfort of the electrode on the skin. They can adapt to different shapes and sizes of skin areas and are more suitable for biopotential acquisition in soft skin areas, such as around the eyes and stomach. The most commonly used electrode on the market is the commercial AgCl electrode [56], whose flat disk structure is easily affected by surface hair when collecting biopotentials. The hair will reduce the contact area between the electrode and the skin, affecting the signal quality. In emergencies, the hair needs to be shaved off with a razor before collecting biopotentials. The unique hook hair structure of the conductive magic tape electrode provides a large gap between the skin and fabric. The hook hair structure of the electrode can go through the hair to collect biopotentials, greatly expanding the application range of the electrode. In addition, the conductive magic tape electrode also has obvious advantages, such as low price, light weight, and easy availability. The enlarged surface of the hook hair is shown in Figure 2a,b. The surface of the Velcro hook hair is coated with a layer of silver particles, which can provide a conductive medium for collecting bioelectric signals. The height of the hook hair is about 2 mm (Figure 2c), which can penetrate most of the sweat and hair on the skin’s surface and has a wide range of application prospects.

### 3.3. Selection of CVE

**Figure 3:**
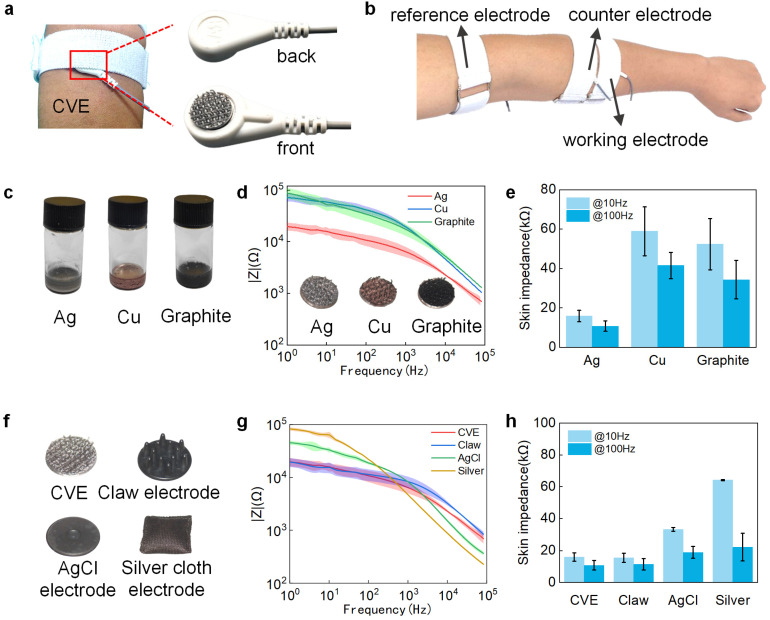
Material selection and performance of CVE with some typical dry electrodes. (**a**) Schematic diagram of CVE inside the elastic strap; (**b**) schematic diagram of arm wearing position when collecting electromyographic signals with CVE; (**c**) materials of silver paste, copper paste, and graphite paste; (**d**) electrochemical impedance curves of electrodes made of silver paste, copper paste, and graphite paste; (**e**) impedance comparison at typical frequencies of 10 Hz and 100 Hz for electrodes made of three pastes; (**f**) pictures of CVE, claw electrode, AgCl electrode, and silver cloth electrode; (**g**) impedance curves of the four electrodes above; (**h**) impedance comparison at typical frequencies of 10 Hz and 100 Hz for these four electrodes.

Low impedance is critical for high-fidelity measures of sEMG because it helps reduce motion artifacts and signal decline. When collecting biopotential signals, the hook surface of the CVE was in direct contact with the skin, and the back was wrapped in a bandage, as shown in Figure 3a. Figure 3b shows the electrode distribution map when using Velcro electrodes to collect biopotential signals. The CVE was assessed with Ag/AgCl electrodes and attached to forearms for EIS measurement. The CVE was used as the working electrode, and one commercial AgCl electrode (counter electrode) was placed at the forearm, while the other commercial AgCl electrode was placed as the reference electrode at the elbow. To develop the best CVE [57], we conducted electrochemical tests on the CVE made of conductive silver paste (left, gray), conductive copper paste (middle, brown), and conductive graphite paste (right, black). From Figure 3d, it can be seen that the electrode made of conductive silver paste has a lower impedance compared to the other two electrodes. At a frequency of 10 Hz, the impedance of the silver Velcro electrode is 73% and 70% lower than that of the copper and graphite Velcro electrode, respectively. The impedance is 74% and 69% lower at 100 Hz, as shown in Figure 3e. Therefore, the silver paste was chosen as the conductive paste for making conductive Velcro electrodes. To demonstrate that the conductive silver Velcro electrode can meet the usage requirements, we conducted impact tests on the CVE, clamp electrode, silver cloth electrode, and conductive AgCl electrode, respectively. The physical images of the four electrodes are shown in Figure 3f. The impedance at 1 Hz to 100 kHz is shown in Figure 3g. In the low-frequency range (below 1 kHz), the CVE exhibits low impedance characteristics comparable to the other electrodes, as shown in Figure 3h. Typically, at 10 Hz, the impedance of the CVE decreased by 52% and 75% compared to the AgCl and silver cloth electrodes, respectively.

### 3.4. Biopotential Acquisition

#### 3.4.1. sEMG Signal Acquisition

**Figure 4:**
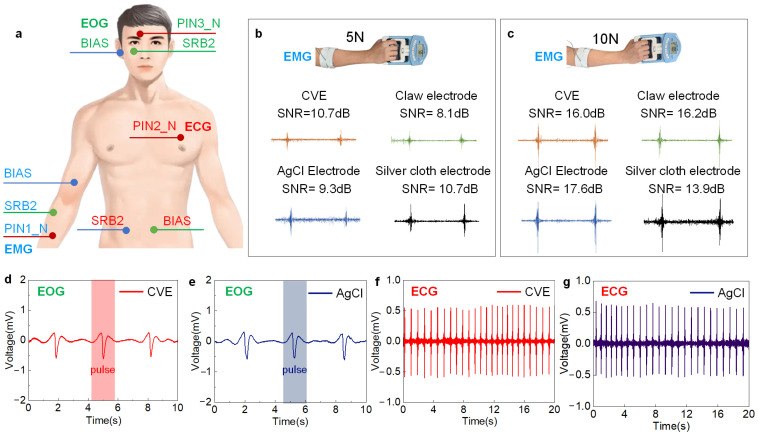
Biopotential acquisition using CVE. (**a**) Distribution of electrode positions for EMG, EOG, and ECG signal acquisition; analysis of SNR of EMG signals collected using CVE, AgCl electrodes, claw electrodes, and silver cloth electrodes under a grip strength of (**b**) 5 N and (**c**) 10 N; EOG signals collected using (**d**) CVE and (**e**) AgCl electrodes, respectively; ECG signals collected using (**f**) CVE and (**g**) AgCl electrodes, respectively.

To validate the advantages of the CVE in electrochemical signal detection, we compared the electromyographic signals collected by the AgCl electrode, clamp electrode, and silver cloth electrode, respectively. As shown in Figure 4a, the placement of the working electrode and counter electrode is designed for the muscles near the arm, including the extensor digitorum, extensor carpi ulnaris, and flexor carpi radialis [58]. Figure 4b,c shows the electromyographic signals collected by four electrodes under grip strengths of 5 N and 10 N, respectively. The CVE electrode exhibits the maximum signal amplitude and minimum noise and motion artifacts. When the grip strength is 5 N, the electromyographic signals collected by CVE show the highest signal-to-noise ratio, which is better than the other three electrodes. When the grip strength increases to 10 N, the signals show comparable quality to the other three electrodes. As the muscle strength increases from 5 N to 10 N, the SNR of the CVE increases from 10.7 dB to 16.0 dB, equivalent to the other three.

#### 3.4.2. EOG Signal Acquisition

We used CVE and AgCl electrodes to collect eye electrical signals, as shown in Figure 4a. The working electrode (CVE/AgCl) was placed on the forehead (1 cm above the eyebrows), the counter electrode (AgCl) was placed 1 cm below the eyes, and the reference electrode (AgCl) was placed on the earlobe. The subjects blinked every 3 s, and the collected eye electrical signals are shown in Figure 4d,e. It can be seen from the figure that the CVE can collect blink signals comparably to AgCl [59]. At the same time, the SNR of the CVE is 19.95 dB, 26.5% higher than the 15.77 dB of the AgCl electrodes. This proves that our electrode has excellent performance in electro-oculography acquisition.

#### 3.4.3. ECG Signal Acquisition

The CVE can be used for high-quality recording of electrocardiogram signals, as well. As shown in Figure 4a, in the electrocardiogram acquisition experiment, CVE and AgCl electrodes were used as working and counter electrodes, placed on the chest and sides of the stomachs of volunteers, and fixed with long bandages. The electrodes are connected to the internally constructed data recording unit through wires, with a sampling rate of 1 kHz, and the recorded data is directly stored in the computer. Using the settings above, we measured the ECG signals of the same volunteer in a relatively short period on the same day using CVE and commercial AgCl electrodes. The data recorded in Figure 4f,g show clear ECG signals from two types of electrodes, where periodic peaks represent different stages of electrical activity during a heartbeat [60]. From the period of the electrocardiogram waveform, it can be estimated that the corresponding heart rate is 75 bpm. In addition, we calculated the SNR of the electrocardiogram signals collected by two types of electrodes. The SNR of the CVE was 25.28 dB, slightly higher than the AgCl electrode of 19.31 dB; the SNR increased by 5.97 dB.

### 3.5. Reliability Testing

#### 3.5.1. Water-Resistance Performance

**Figure 5:**
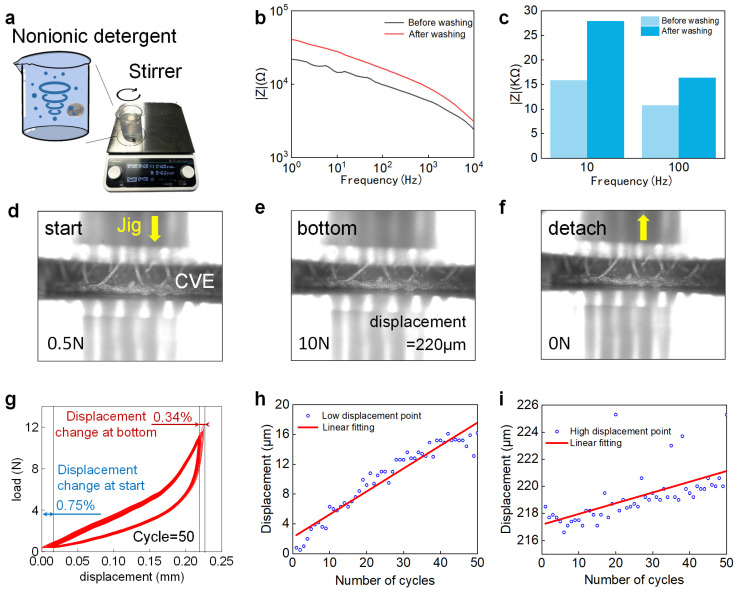
Water resistance and compression resistance tests. (**a**) Magnetic stirrer with stirring function; (**b**) impedance comparison of conductive Velcro before and after washing; (**c**) comparison of impedance before and after washing conductive Velcro (10 Hz and 100 Hz); (**d**) when the load is 0.5 N, the schematic diagram of compression Velcro; (**e**) when the load is 10 N, the schematic diagram of compression Velcro; (**f**) Velcro photo after cyclic compression ends; (**g**) after 50 cycles of compression, the corresponding relationship between displacement and load; (**h**) the lowest displacement change diagram during cyclic compression; (**i**) the highest displacement change diagram during cyclic compression.

Waterproof performance can be tested by ultrasonic [61] or stirring [62] methods; we chose the latter. The CVE was washed with 4 g/L nonionic detergent at 40 °C for 30 min according to the standard method (ISO 105-C06-A1S, 2010), as shown in Figure 5a. Then, the electrochemical performance of the washed CVE was verified by measuring the impedance of the conductive Velcro before and after washing using an electrochemical workstation [63]. The experimental results show that the impedance of the conductive Velcro after washing increased from 15.88 kΩ to 27.9 kΩ at 10 Hz, with an increase of 75% in impedance, but still lower than that of the AgCl electrode (33.26 kΩ) and the silver cloth electrode (64.16 kΩ). The impedance at 100 Hz increased from 10.77 kΩ to 16.4 kΩ, with an increase of 52%, still lower than that of the AgCl electrode (18.84 kΩ) and the silver cloth electrode (22.12 kΩ). This proves that the electrode has excellent waterproof performance.

#### 3.5.2. Compression-Resistance Performance

It is very important that the Velcro electrode can still return to its original shape after multiple uses. To verify the elasticity and long-term durability of the hook hairs in the Velcro electrode, we conducted the following experiments. Using a mechanical stretching platform to repeatedly compress the hook hairs of the conductive Velcro electrode, we set the load range to 0.5 N to 10 N, and the position of the indenter continued to decline (the load gradually increased from 0.5 N), as shown in Figure 5d. When the load increased to 10 N, the position of the indenter continued to rise (the load gradually decreased from 10 N), as shown in Figure 5e. When the load decreased to 0.5 N, it was defined as one cycle. After 50 cycles of repeated testing, it can be seen that the shape of the Velcro after repeated compression is almost the same as before compression (Figure 5f); the experimental results are shown in Figure 5g. It can be seen that there is not much change in displacement during the cyclic test, and the lowest point of displacement of the pressure head shows an upward trend during the experimental process. After 50 repeated compressions, the lowest point of displacement rose by 15.4 μm, and the highest point of displacement rose by 6.8 μm, both of which are less than 1% of the total height (2 mm) of the conductive Velcro hook hair. Therefore, it can be proved that our CVE hook hair can still recover to its original position after multiple uses.

### 3.6. sEMG-Based Gesture Recognition

**Figure 6:**
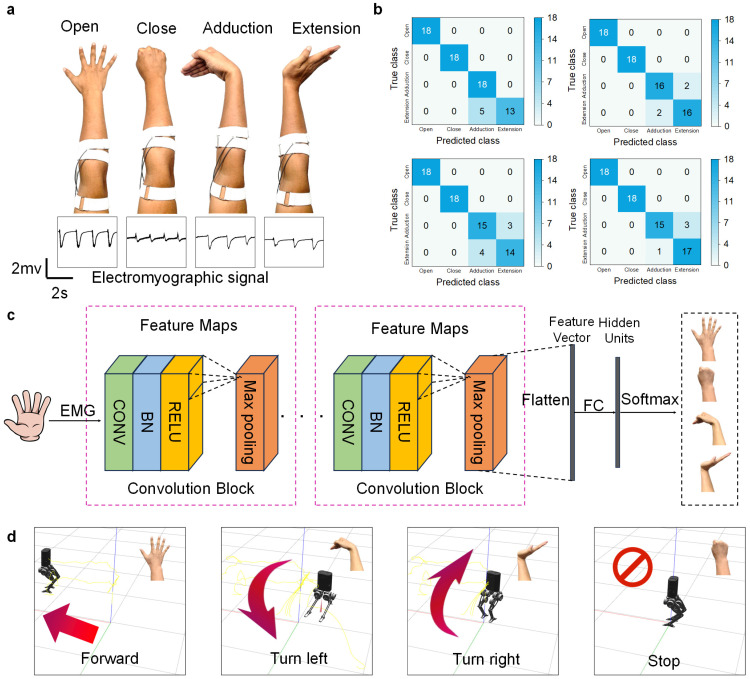
CNN convolutional neural network for gesture recognition by EMG signals recorded from CVE. (**a**) Four hand gestures to be classified: palm open, palm closed, wrist adduction, wrist extension; (**b**) confusion plots for all the gesture recognition results; (**c**) internal structure diagram of convolutional neural network; (**d**) robot moves forward, turns left, turns right, and stops in the virtual environment controlled by the EMG signals.

In this expense measure, four commonly used artifacts were used as verification artifacts. The artifact diagram is shown in Figure 6a. When collecting electrochemical signals, an artifact was made every two seconds, and each artifact was measured 60 times. A total of 42 data points were used as the training sets to train the convolutional neural network. After the neural network was trained, the remaining 18 sets of data were used as the test sets to verify the accuracy of gesture recognition. The test results are shown in Figure 6b. After four repeated tests, the gesture recognition accuracy rates were 93.1%, 94.4%, 90.3%, and 94.4%, respectively [64]. Even after 100 repeated tests, the gesture recognition accuracy rate still reached over 90%. The neural network architecture is shown in Figure 6c, where there are two convolutional blocks in total, each including a CONV layer and a BN layer. The RELU layer and MAX pooling layer were trained with a learning rate of 0.001. To apply the gesture recognition results to our daily lives, we used gesture control to control the ROS robot system. First, we loaded a robot on the simulation interface, and the control rule was that the robot moved forward with the hand open, turned left with hand adduction, turned right with extension, and paused with the hand closed. After training, we were able to use gestures to control the robot in the simulation system to move, and the accuracy of the robot executing instructions reached over 90% [65], as shown in Figure 6d. The experimental results show that the electromyographic signals collected using CVE could accurately recognize four types of gestures and have broad application prospects in the future [66,67].

## 4. Conclusions

We developed a conductive Velcro electrode with elastic hook hair microstructures. The electrode uses an innovative and concise fabrication method to obtain conductivity. We demonstrated CVE use for high-quality ECG and EMG signal recording applications. The electrode shows excellent properties in impedance, signal-to-noise ratio, water resistance, and mechanical resistance, and is suitable for different parts of the body with or without hair. The CVE has also shown great potential in gesture recognition applications, which can accurately recognize multiple gestures, providing new ideas for scenarios that require gesture classification. Compared to other dry electrodes, Velcro is sourced from a variety of raw materials, with advantages such as low cost, simple to manufacture, and well-suited for large-scale production. In the future, we will continuously optimize the microstructure of the hook and verify the EEG acquisition effect in hairy areas to better collect bioelectric signals through hair. The Velcro dry electrode can be widely used in areas of human–machine interaction, wearable healthcare monitoring, myoelectric prostheses, etc.

## Data Availability

Data are contained within the article.
